# *Marrubium vulgare* L. Leave Extract: Phytochemical Composition, Antioxidant and Wound Healing Properties

**DOI:** 10.3390/molecules22111851

**Published:** 2017-10-28

**Authors:** Bédis Amri, Emanuela Martino, Francesca Vitulo, Federica Corana, Leila B. Ben-Kaâb, Marta Rui, Daniela Rossi, Michela Mori, Silvia Rossi, Simona Collina

**Affiliations:** 1 Département de Biologie, Faculté des Sciences de Tunis el Manar, Unité de recherche ‘Nutrition et métabolismes azoté et protéines de stress’ (99 UR/09-20), 1002 Tunis, Tunisia; bedisamri@gmail.com (B.A.); leila.bk@planet.tn (L.B.B.-K.); 2 Department of Earth and Environmental Sciences, University of Pavia, Via S. Epifanio 14, 27100 Pavia, Italy; 3 Centre for Health Technologies (CHT), University of Pavia, Viale Taramelli 12, 27100 Pavia, Italy; silvia.rossi@unipv.it; 4 Indena S.p.A., Via Don Minzoni, 6, 20090 Settala, Italy; francesca.vitulo@indena.com; 5 Centro Grandi Strumenti (CGS), University of Pavia, Via Bassi 21, 27100 Pavia, Italy; federica.corana@unipv.it; 6 Department of Drug Sciences, Medicinal Chemistry section, University of Pavia, Viale Taramelli 12, 27100 Pavia, Italy; marta.rui01@universitadipavia.it (M.R.); daniela.rossi@unipv.it (D.R.); michela.mori@unipv.it (M.M.)

**Keywords:** wound healing, *Marrubium vulgare* L. leave extract, q-NMR, antioxidant properties, HPLC-UV/PAD MS analysis

## Abstract

Several factors contribute in wound generation, e.g., accidental traumas or surgery, and in certain cases, this dermal injury may have a devastating outcome. When skin damage occurs, the human body puts in place a sophisticated choreography, which involves numerous repairing processes to restore physiological conditions. Nevertheless, natural healing mechanisms are ineffective towards chronic or non-healing wounds and thus, therapeutic strategies may represent the only beneficial alternative to counteract these tissue insults. Over the years, numerous studies showed the great potential of plants in promoting wound healing, by virtue of their high contents in antioxidant species. These compounds trigger a molecular cascade that collimate into the promotion of reparative processes. In this article, we report on the potential effect on wound healing of *Marrubium vulgare* L., a medicinal plant well known for several pharmaceutical activities. To this aim, the methanolic extract was prepared and subjected to a phytochemical investigation, quantifying the amount of marrubiin via NMR and drawing the phytochemical fingerprint via high performance liquid chromatography—ultra violet/photodiode-array detection-electrospray/mass (HPLC-UV/PAD-ESI/MS) analysis. Lastly, the antioxidant properties and wound healing potential have been evaluated.

## 1. Introduction

The plant kingdom is a precious source of healing remedies for various pathologies. To date numerous research groups direct their efforts towards the identification of natural medicinal products and plant extracts able to prevent or treat different pathological manifestations [1]. As reported by the World Health Organization (WHO), approximately 80% of people worldwide exploit traditional medicine for their health care [2]. In this context, the WHO launched the Traditional Medicine Strategy 2014–2023 (WHA62.13). The strategy aims to develop action plans that will strengthen the role that traditional medicine plays in keeping populations healthy [3].

Plant-based strategies have been widely used for wound healing and skin regeneration and, their therapeutic application dates back to the ancient times [4]. From a physiopathological standpoint, wound is an injury that damages the dermal layer of the skin and the natural process, which leads to restore the structural and functional integrities of injured tissues, represents the wound healing [5]. Accidental traumas or surgery are the main causes of skin damage, however this event may occur as a consequence of other pathologies, i.e., cancer or diabetes [6,7]. Accordingly, the human body promotes a repairing “choreography”, involving several biochemical and cellular pathways, in order to repair the lesions and to restore the physiological conditions [8]. In this context, fibroblasts play a pivotal role, regulating the synthesis of some proteins, among them the most representative is collagen, being essential in the process of healing throughout the tangled repairing mechanism [9]. Nevertheless, the impairment of this sophisticated repairing process leads to chronic or non-healing wounds, which may result in severe clinical complications or even patient death [10]. The failure of wound healing process may be caused by deficiencies in nutrition factors (i.e., vitamin A or C), which are essential in cellular differentiation, immune function and collagen formation [11]. Additionally, several studies support the crucial role of nitric oxide and reactive oxygen species (ROS) in regulating healing [12]. In fact, high concentrations of both Oxygen and Nitrogen-centered reactive species are present in wound sites [13]. These substances can induce harmful effects on cells and tissues and even promote oxidative stress that generates lipid peroxidation, damage of DNA and enzyme inactivation, including free-radical scavenger enzymes [10]. Furthermore, reparative mechanisms may stimulate the production of antioxidants in wound site, providing a suitable environment for tissue repairing [14]. In light of these considerations, antioxidants have a crucial role in wound healing process. Compounds with free-radical-scavenging properties promote the proliferation of fibroblast adhesion during injury healing, stimulating re-epithelization, neovascularization and maturation of extracellular matrices [4,13]. Therefore, molecules endowed with antioxidant and antimicrobial properties may represent potential therapeutic tools to enhance and accelerate wound healing process [5]. Several natural compounds, i.e., flavonoids, triterpenes, alkaloids and polyphenols show antioxidant and antimicrobial effects and are able to promote one or more mechanisms of the reparative process [6]. Accordingly, numerous plant extracts have been employed to promote wound healing with a high degree of success, leading in some cases to the disease remission [15]. An interesting example is represented by *Termalia* species, used in Ayurvedic medicine, showing beneficial effects against wounds. This property has been associated with tannic acid that stabilizes collagen and elastin in the extracellular matrix [16]. Moreover, the ability of *Centella asiatica* in improving the healing of small wounds, hypertrophic scars and burns is widely known [17].

The current work is part of this scenario and it aimed at studying the potential of *Marrubium vulgare* L. (Horehound Lamiaceae) in wound healing process. This grey-leaved herbaceous perennial plant is native to Eurasia and northern Africa zones. It is traditionally employed against respiratory infections such as bronchitis, coughs and asthma as already mentioned in first text concerning medical knowledge in the Roman world: *De Medicina* [18,19]. Nevertheless, literature data show that other pharmacological activities can be ascribed to Horehound, including antihypertensive, analgesic, anti-inflammatory, hypoglycemic, vasodilator and antidiabetic properties [20,21,22,23,24,25,26]. Moreover, an interesting antibacterial activity against both Gram-positive and Gram-negative bacteria [27], as well as strong antioxidant behavior [28,29,30] were evaluated. In summary, the therapeutic properties of *Marrubium vulgare* are extensively described and its medical preparations are spread all over the world. The main pharmacological activities of *Marrubium vulgare* have been associated to marrubiin, a bitter lactone considered as the genus chemotaxonomic marker [14]. Amino acids, polysaccharides and tannins are the major constituents of buds, flowers and fruits [31], whereas high amounts of phenols and flavonoids are mainly localized in leaves [29]. Considering that phenols and related compounds may improve the proliferation of fibroblasts and the migration of cells, thus promoting the formation of new blood vessels and capillaries [25,32], in the present study we focus our attention on *Marrubium vulgare* L. leaves. Based on our experience, we prepared a hydroalcoholic leave extract (HA-LE) applying a Microwave Assisted Solvent Extraction (MASE), a methodology suitable for preparing high-values bioactive extracts [33,34,35]. The content of phenols and flavonoids was evaluated via colorimetric analysis and the amount of marrubiin determined by quantitative Nuclear Magnetic Resonance (q-NMR). Moreover, its phytochemical profile was drawn via high performance liquid chromatography—ultra violet/photodiode-array detection-electrospray/mass (HPLC-UV/PAD-ESI/MS) analysis. To determine its therapeutic potential, antioxidant properties and wound healing activity were investigated. Altogether, this work lay the foundation for further scientific studies.

## 2. Results

### 2.1. Extract Preparation

According to the data reported in literature, the air-dried leaves of *Marrubium vulgare* L. were extracted with a solution of 80/20 methanol (MeOH)/water (*v*/*v*) [24]. Basing on our experience in this field [33,34,35,36,37], we set up a microwave-assisted solvent extraction (MASE) procedure. Briefly, the plant material was extracted with a 80/20 MeOH/water solution (*v*/*v*) in a multimode (for processing bulk materials) microwave apparatus at 60 °C for 10 min with a power of 800 W. The extract was separated by Buchner filtration, the so-obtained crude extract was treated with charcoal to remove the chlorophyll and then with polivinylpirrolidone (PVP) to take out tannins, which can alter the results of biological assays [38].

### 2.2. Phytochemical Analysis

#### 2.2.1. Preliminary Screening

Preliminary phytochemical analysis of the extract was performed via Thin Layer Chromatography (TLC). The TLC results, obtained through the employment of specific mobile phases and revelation reagents showed the presence of polyphenols, flavonoids and naphthoquinones, whereas coumarins, alkaloids, and saponins were not detected [39]. In light of these preliminary results total phenolic and flavonoid contents (TPC and TFC, respectively) were determined, in order to evaluate the amount of the main antioxidant components of *Marrubium vulgare* HA-LE. TPC was determined measuring the absorbance of the extract solution, reacted with Folin–Ciocalteu reagent, in comparison with the absorbance of a standard solution of gallic acid equivalents. This experiment suggests that the phenolic component is present in a good amount in aqueous methanol extract. Conversely, TFC was measured and compared with a standard solution of (+)-catechin. The spectrophotometric results lead to assume that flavonoids are the main antioxidant components of our extract. TPC resulted 6.02 ± 0.01, expressed as mg gallic acid equivalents (GAE) per g dry weight (DW), whereas TFC 45.21 ± 0.01, expressed as mg catechin equivalents (CE)/g DW (mg CE/g DW). The obtained results confirmed that the extract is rich in phenols and flavonoids, and these data led us to pursue with our studies aimed at underlining the potential role of our extract in promoting wound healing effect.

#### 2.2.2. Marrubiin Content Determination

**Figure 1:**
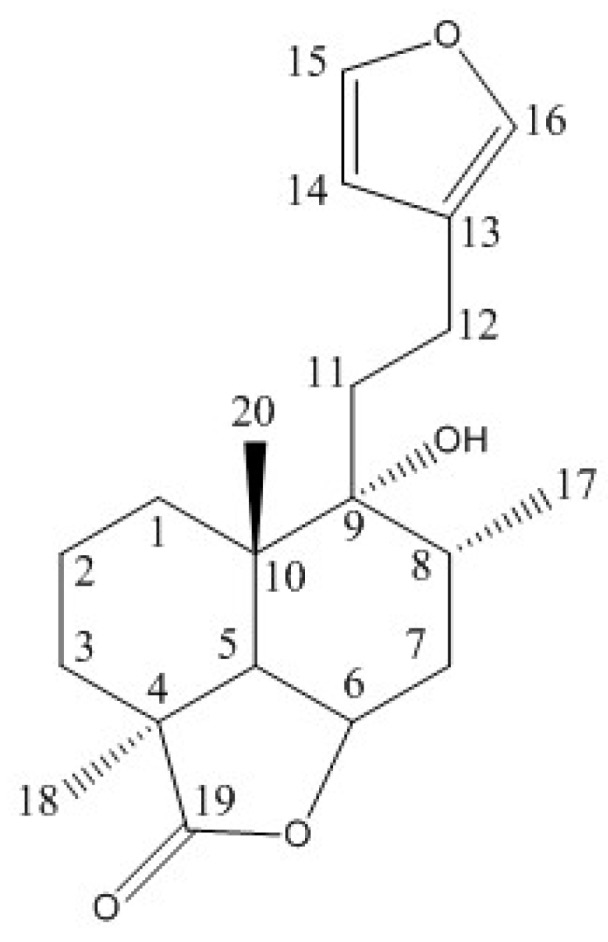
Marrubiin structure.

**Figure 2:**
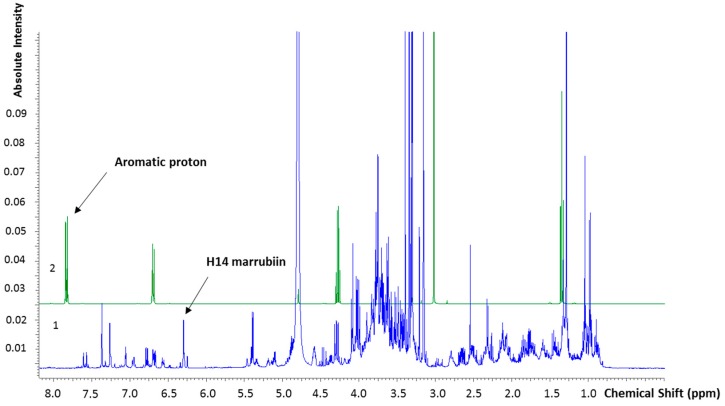
Superimposition between (1) Ethyl-4(dimethylamino) benzoate and (2) *Marrubium vulgare* hydroalcoholic leave extract (HA-LE).

**Figure 3:**
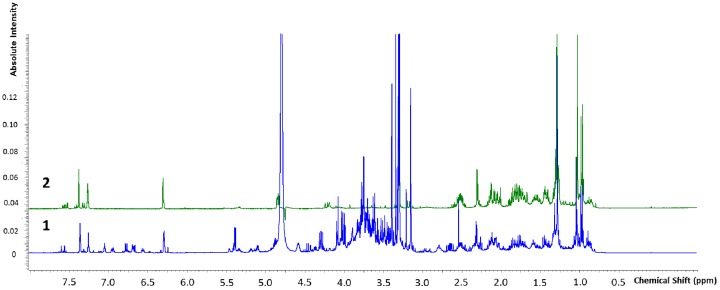
^1^H spectrum superimposition of (1) *Marrubium vulgare* HA-LE and (2) marrubiin reference standard.

We quantified marrubiin (Figure 1), which is considered a chemotaxonomic marker for the genus *Marrubium* (Lamiaceae) [14]. To this aim, we adopted a q-NMR approach, a suitable methodology for determining the content of the prominent compound in vegetal matrices [40,41,42]. Firstly, the NMR analysis of marrubiin (reference standard) in Methanol-D4 was performed and the signals assigned by comparing ^1^H, ^13^C, COSY and HSQC spectra with literature data [43,44] (for spectra see Supporting Information). The chemical shifts (δ (ppm)) are reported in the Table 1. At the meantime, the ^1^H-NMR spectrum of HA-LE was recorded and the signals belonging to the marrubiin identified. In doing q-NMR assays the reference standard has to be evaluated against an internal standard (I_Std_) endowed with known purity, molecular mass and weight. The I_Std_ has to fulfill some other pivotal requirements: (i) well resolved proton signals; (ii) the absence of overlap with the peaks characterizing the molecule of interest. To quantify marrubiin in HA-LE, ethyl-4(dimethylamino)benzoate has been selected as I_Std_ for its well-defined aromatic proton resonance at 7.83 ppm, which is separated from all signals of the *Marrubium vulgare* HA-LE, as disclosed in the superimposition reported in Figure 2. As evidenced in Figure 3, where the overlapped NMR spectra of HA-LE (1) and marrubiin standard (2) are shown, the signal at 6.31 ppm is one undisturbed signal of marrubiin, unequivocally assigned to the H14. Thus, it was selected as reference peak, suitable for conducting the subsequent q-NMR study.

Once the reference signals have been identified, we pursued with the quantitative analysis. The I_Std_ ethyl-4(dimethylamino)benzoate and the extract were weighed and dissolved in Methanol-D4, obtaining two I_Std_ mother solutions of 7.2 and 7.4 mg/mL and the HA-LE of 30 mg/mL. The final solutions were prepared by adding 0.5 mL of each I_Std_ solution to the extract (0.75 mL), thus obtaining a final volume of 1.25 mL. To avoid experimental errors, before performing quantification experiments, the T1 relaxation time of the nuclei of interest was determined, as suggested by Malz et al. [44]. Based on the measurements, we set relaxation delay at 30 s. The ^1^H spectra were then recorded (without solvent suppression) and the previously selected signals (at 6.31 ppm for marrubiin and 7.83 ppm for ethyl-4(dimethylamino)benzoate) were integrated. The integral values were then employed to calculate the purity (P_x_) of marrubiin in the extract [44]. Based on the ratio of intensities, considering the number of nuclei contributing to the resonance (N_x_, N_Std_), the molar masses (M_x_, M_Std_) and initial weight of internal standard (considering its relative content P_Std_) and analyte, the relative content of marrubiin (principal component, P_x_) was calculated as *w*/*w* % using the following equation:

$$P_{X}=\frac{I_{X}}{I_{\mathrm{Std}}}\frac{N_{\mathrm{Std}}}{N_{x}}\frac{M_{X}}{M_{\mathrm{Std}}}\frac{m_{\mathrm{Std}}}{m_{x}}P_{\mathrm{Std}}$$

On the basis of the relative integral ratios, the content of marrubiin in the sample was determined as 6.62% *w*/*w*, with an RSD of 0.26% between the two prepared solutions.

#### 2.2.3. HPLC-UV/PAD MS Analysis

**Figure 4:**
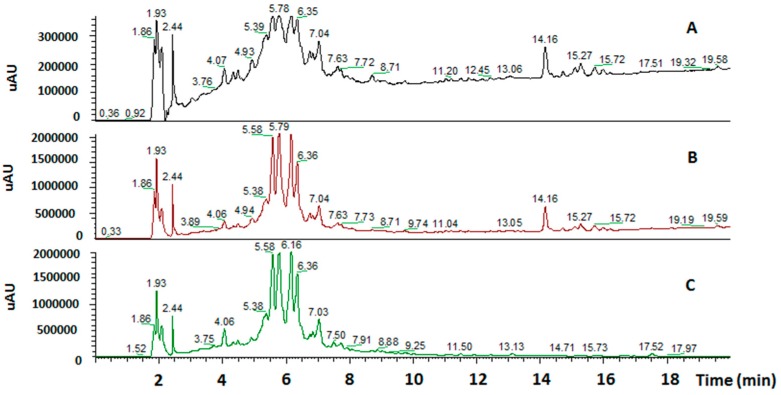
HPLC-UV chromatograms of *Marrubium vulgare* HA-LE. Detection UV at 220 nm (**A**), 254 nm (**B**) and 280 nm (**C**).

The crude extract was analyzed to draw the phytochemical components through high performance liquid chromatography using an ultraviolet photodiode array detector (HPLC-UV/PAD). A meticulous analytical screening was performed to achieve a suitable separation of the components in the extract. Several columns (Kromasil C-18, Chromolith Performance RP-18 endcapped, Discovery Cyano and XBridge Phenyl) were tested, under different elution conditions [MeOH, acetonitrile (ACN) and water], adopting several gradient modes and in all cases 0.1% of acid (acetic or trifluoroacetic or formic acids) was added to the mobile phase. Furthermore, the chromatograms were recorded at different wavelengths (220, 254, and 280 nm). The best results in terms of peak resolution, peak shape and time of analysis were achieved using an XBridge Phenyl (5 µm, 4.6 × 150 mm) column in gradient elution conditions, using water and ACN with formic acid. These analytical conditions led us to obtain a satisfactory separation of all components in 20 min (Figure 4). 

**Figure 5:**
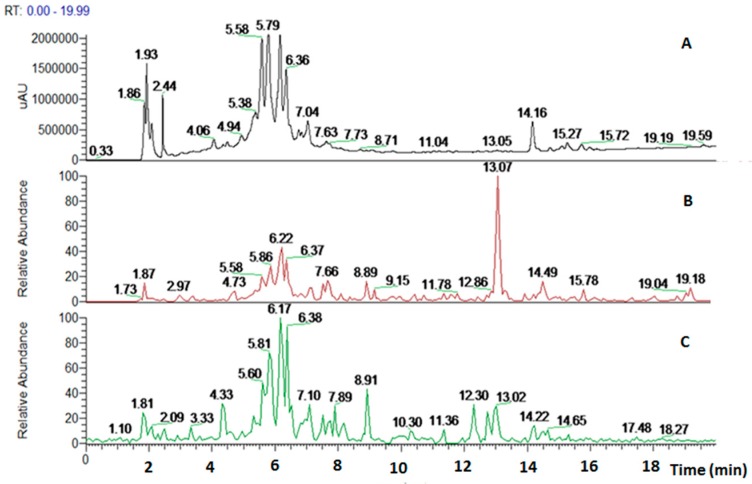
HPLC-UV chromatograms of *Marrubium vulgare* HA-LE. Detection UV at 254 nm (**A**); reconstructed ion chromatograms (RIC) positive ions (**B**); RIC negative ions (**C**).

The *Marrubium vulgare* L. extract was analyzed through HPLC-UV/PAD-ESI/MS. The HPLC-UV chromatogram (λ = 254 nm) together with the reconstructed ion chromatograms (RIC) in both positive and negative ion mode is shown in Figure 5.

The ESI-MS of the main peaks allowed the determination of the molecular weights (Table 2). The peak at 5.58 min may be attributed to marruboside, a phenylethanoid glycoside, recently identified in *Marrubium vulgare* by comparison of its fragmentation with literature data [45]. In details, negative fragment ions correspond to the loss of a pentose unit (apiose) and of a caffeoyl moiety, whereas the MS/MS spectrum in positive mode evidenced the presence of three fragment ions (*m*/*z* 624.63, 478.60 and 324.92) due to the sequential loss of two apiose, one rhamnose and one hydroxytyrosol units (See Supporting Information). Compounds with t_R_ of 5.79, 6.17 and 6.36 min showed a ESI/MS/MS fragmentation behavior similar to that of marruboside. Based on the MS/MS negative and positive ions data, the peaks at t_R_ 5.79 min and 6.36 min may be attributed to forsythoside B and to verbascoside, respectively [46,47,48]. The MS/MS spectrum in positive ions mode of compound with t_R_ 6.17 min showed the loss of a fragment of 132 Da, suggesting the presence of the pentose unit (See Supporting Information) of samioside [48].

**Figure 6:**
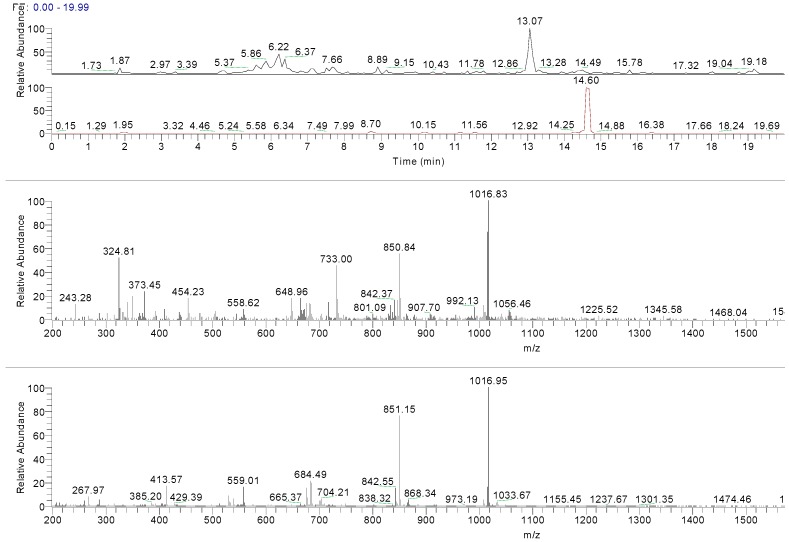
From top to bottom RIC of *Marrubium vulgare* HA-LE, RIC of marrubiin standard solution, ESI-MS spectrum of peak at t_R_ 14.49 in *Marrubium vulgare* HA-LE, ESI-MS spectrum of marrubiin standard.

Two additional peaks—undetected in UV mode—at t_R_ 13.07 and 14.49 min were recorded in RIC chromatograms (positive ion experiments). The first peak could be attributed to the deacetylvitexilactone, in virtue of its pseudo molecular ion at *m*/*z* 366.6 [M + H]^+^ and its major fragments in positive mode (*m*/*z* 319, 301 and 289) (Table 2) [48,49,50]. The second compound with a t_R_ of 14.49 min is related to marrubiin (molecular weight 332 Da), as confirmed by the analysis of marrubiin standard (Figure 6). The ESI-MS spectra of marrubiin—present in both the standard solution and the extract—match, showing an intensive peak at *m*/*z* 1016 corresponding to ion [6M + H + K]^2+^. The results are summarized in Table 2.

### 2.3. Biological Investigation

#### 2.3.1. Antioxidant Activity

The extract free-radical scavenging (FRS) activity *Marrubium vulgare* HA-LE was determined by 2,2-diphenyl-1-picrylhydrazyl (DPPH) assay, using a green tea standardized extract (Greenselect^®^, Indena S.p.a) as standard (EC_50_ = 4.6 μg/mL). The stock solution of *Marrubium vulgare* HA-LE (250 µg/mL) in MeOH was serially diluted into a range of 2.5–120 µg/mL, added to a methanolic solution of DPPH and the absorbance of the resulting solution evaluated. The antioxidant activity of *Marrubium vulgare* HA-LE solution at different concentrations was calculated by determining the decrease in the absorbance. Our crude extract showed an EC_50_ of 38.56 ± 0.10 μg/mL.

#### 2.3.2. Activity on Fibroblast Proliferation

**Figure 7:**
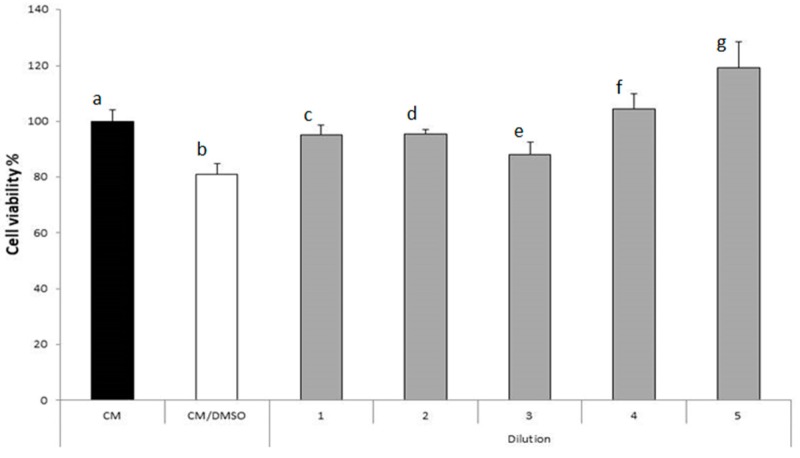
Cells viability percentage (%) of different dilutions of *Marrubium vulgare* HA-LE (means ± s.d.; *n* = 8); Anova one way (*p* < 0.5): a vs. b/e/g; b vs. c/d/f/e/g; c vs. g; d vs. g; e vs. f/g; f vs. g. Dilution: **1** 500 µg/mL; **2** 250 µg/mL; **3** 125 µg/mL; **4** 50 µg/mL; **5** 5 µg/mL.

Cytocompatibility of *Marrubium vulgare* HA-LE on human dermal fibroblast (HDFs) cell line was assessed by MTT [3-(4,5-dimethylthiazol-2-yl)-2,5-diphenyltetrazolium bromide-tetrazolium dye] method. In Figure 7, cell viability percentages of human dermal fibroblast (HDFs) upon contact with different dilutions (into a range of 500–5 µg/mL) of *Marrubium vulgare* HA-LE are reported. It can be observed that all the dilutions are characterized by % cell viability values higher than that obtained for the complete medium (CM) mixed with DMSO, at the same concentration present in the extract. Such results indicate that the extract components, at all the considered dilutions, decrease the cytotoxicity of DMSO. Moreover, at the concentration of 5 µg/mL a % cell viability higher than that of CM was observed; it indicates that such sample has the capability to improve cell proliferation.

**Figure 8:**
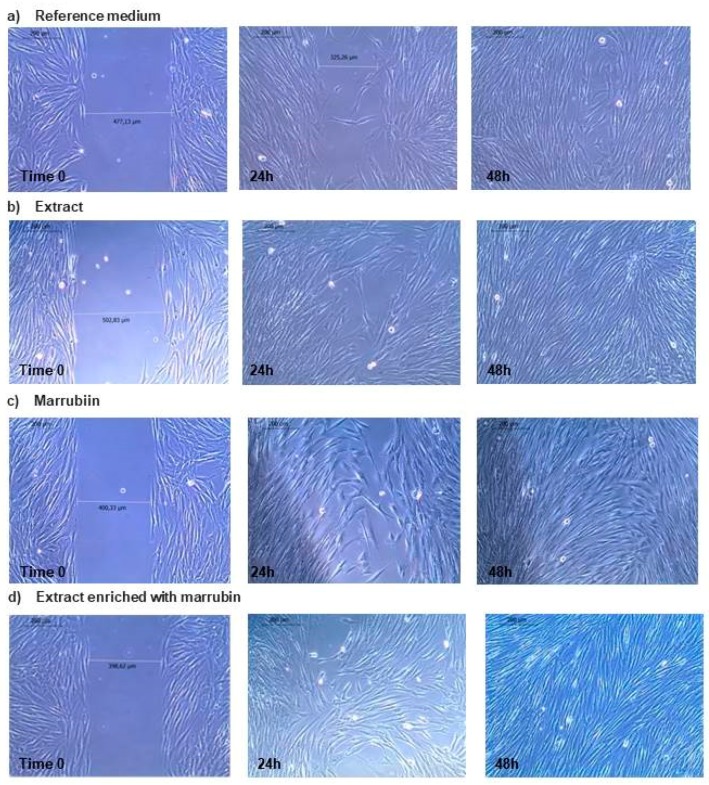
Photographs of fibroblasts grown in the gap after contact for different times with: (**a**) reference medium; (**b**) *Marrubium vulgare* HA-LE (5 µg/mL); (**c**) marrubiin (5 µg/mL); (**d**) *Marrubium vulgare* HA-LE (5 µg/mL) enriched with marubbin (5 µg/mL).

Considering that dilution **5** is able in promoting the cell viability, the concentration 5 μg/mL was selected to pursue with the subsequent wound healing assays. In Figure 8, the pictures of fibroblasts grown in the gap after contact with extract for increasing times (0, 24 and 48 h) are reported. Marrubiin (5 µg/mL) was also tested. As reference, the medium employed for sample dilution was considered. At time 0, the gap between cells is quite well visible for all the samples. In the case of the extract, after 24 h cells are sub-confluent and after 48 h the complete confluence is reached. To understand if the effect is due to marrubiin, an additional experiment was performed. Marrubiin was tested at the same concentration of the extract (5 μg/mL): after 24 h a behavior similar to the extract was evidenced, but after 48 h a complete cell confluence was not reached. In order to better clarify if marrubiin has a role in wound healing properties of HA-LE, an extract enriched with marrubiin was also tested (Figure 8d). The so-obtained extract does not produce any improvement in sample effect on fibroblast proliferation.

The results obtained indicate that the extract is characterized by wound healing properties and it is worth noting that its effect is not related only to marrubiin.

## 3. Discussion

The plant kingdom is an inestimable source of phytochemicals useful for treating several pathological conditions. Among the plethora of plant-based medical extracts, throughout this work we showed the therapeutic potential of *Marrubium vulgare*, which is currently involved in numerous medical preparations, endowed with anti-inflammatory, antidiabetic and antiasthmatic activities, as well as in cosmetic applications [18,19,20,21,22,23,24,25,26]. With the aim to evaluate the wound healing potential/properties of the *Marrubium vulgare* leaves, we prepared a HA-LE adopting a MASE-based methodology. The so-obtained crude extract was subjected to a treatment with charcoal and PVP to remove the chlorophyll and tannins, respectively [38]. The HA-LE was then evaluated via a preliminary TLC analysis, adopting different mobile phases and revelation reagents. This study suggested that *Marrubiun vulgare* HA-LE contains flavonoids and phenolic compounds, according to literature data [29]. Therefore, TPC and TFC were determined through UV-method and, they resulted of 6.02 ± 0.01 mg/g (GAE/DW) and 45.21 ± 0.01 mg/g (CE/DW), respectively. 

To better characterize our extract, the amount of marrubiin, a diterpenoid lactone considered as the chemotaxonomic marker for the genus [51,52], was determined. At this purpose, q-NMR was selected as fruitful method, since it do not require sample manipulations and allows in one step the proof of identity and the content evaluation of marrubiin. The percentage of marrubiin measured in our HA-LE (6.62%) suggests that our extractive protocol is a valuable and productive alternative to the conventional extraction methods discussed so far (0.4%, 0.1% and 0.12% respectively for Popoola et al., (2013) [43]; Stulzer et al., (2006) [22] and Rodrigues et al., (1998) [53]).

To generate a phytochemical fingerprint of the extract a HPLC-UV/PAD-ESI/MS analysis was performed, according to our previous experience [54]. Firstly, an analytical screening was set up to identify the suitable conditions aimed at separating the extract components. The best resolution method revealed the presence of several phenylethanoid glycoside compounds (retention times: 5.58, 5.79, 6.17 and 6.36 min), which have been identified by comparison with literature data. These metabolites are characterized by a sugary core (beta-glycopyranose) linked to a benzoic acid unit and a phenylethyl ring, via ester or glycosidic bond. Moreover, deacetylvitexlactone and marrubiin compounds, belonging to the labdane-type diterpene family, were detected through RIC chromatograms (positive ion experiments), since they are undetected in UV mode. It is worth noting that phenylethanoid glycoside compounds have been correlated with antioxidant properties, antibacterial, neuro- and radio-protective and anticancer effects [55]. Of particular interest is the antioxidant behavior, since antioxidant compounds may have beneficial effect in promoting reparative mechanisms when an injury occurs. Exogenous compounds, in synergy with the endogenous molecules, may promote the restore of physiological conditions. Lastly, phenylethanoid compounds gained relevance in the skin-care industry and thus, they are becoming useful phytochemicals to develop botanical products, endowed with wound healing properties [47]. 

As discussed before, the HA-LE is characterized by a relevant content of flavonoids and phenols, which are related to tissue repair properties. In fact, literature evidences suggest that both flavonoids and phenols promote the fibroblast proliferation and the cell migration up to the formation of new blood vessels and capillaries [18,22]. Moreover, HA-LE showed a good FRS activity, with an EC_50_ of 38.56 ± 0.10 μg/mL (DPPH assay). Our extract possesses higher antioxidant effect than *Marrubium* *peregrinum* extract (EC_50_ of 187.41 μg/mL) [56]. These data may be attributed to the richness of HA-LE in phenylethanoids and flavonoids, which guarantee to the extract a potential activity in reducing ROS and in promoting wound healing process, since ROS are produced in high amount at the injured site [29,57]. Taken together these evidence prompted us to evaluate the wound healing potential of the extract. A preliminary MTT test evidenced that at 5 μg/mL concentration, the *Marrubium vulgare* HA-LE is non-cytotoxic and able to improve fibroblast growth. This capability was subsequently confirmed by the results of in vitro wound healing test. Interestingly, the HA-LE added with a further amount of marrubiin (about 3%) showed the same cell proliferation properties of HA-LE as such. On the bases of these results, we hypothesized that marrubiin do not play a key role in the wound healing process. This result was confirmed by testing marrubiin at a concentration of 5 μg/mL, in fact the compound promotes a lesser fibroblast growth at 48 h compared to the HA-LE. 

In conclusion, we found that *Marrubium vulgare* HA-LE may be effective against skin injuries, and this activity is marrubiin-independent. Our ongoing researches are addressed to isolate the main secondary metabolites with the final aim to identify compounds endowed with wound healing properties.

## 4. Materials and Methods

### 4.1. Chemicals and Reagents

All chemicals and reagent were purchased from Carlo Erba (Milano, Italy) and were of the highest purity available. 2,2-diphenyl-1-picrylhydrazyl radical (DPPH), polyvinylpyrrolidone (PVP) and charcoal were obtained by Sigma Aldrich (Milan, Italy). 

For NMR quantification, reference standard marrubiin was obtained by Carbonsynth, ethyl-4(dimethylamino)benzoate was purchased by Fluka analytical (ethyl-4(dimethylamino)benzoate, standard for quantitative NMR, traceCERT^®^, potency 99.85%). All the spectra were recorded in Methanol-D4, 99.98% purity (Sigma Aldrich, Milan, Italy).

### 4.2. Instrumentation

Microwave-assisted solvent extraction (MASE) was performed on multimode microwave apparatus using a closed-vessel system (MARSX press, CEM Corporation, Matthews, NC, USA).

HPLC-UV/PAD-CD analyses were performed on a Jasco system (Jasco Europe S.r.l., Cremella, LC, Italy) equipped with a Jasco AS-2055 plus autosampler, a PU-2089 plus pump and a MD-2010 plus multi-wavelength detector coupled to a CD-2095 plus circular dichroism detector. The HPLC-ESI-MS analyses were carried out on ThermoFisher Scientific HPLC/UV/MS system (Thermo Scientific LCQ FLEET ion trap mass spectrometry) controlled by XcaliburTM Software Version 2.1 (Thermo Fisher Scientific, Waltham, MA, USA).

The absorbance was measured by a UV-Visible spectrophotometer (Lambda 25 UV/VIS spectrometer, Perkin Elmer Instruments, Waltham, MA, USA).

### 4.3. Plant Growth

Seeds were issued from non-contaminated wild population in the region of Béja (Northwestern Tunisia; latitude 36°43′30″ (N), longitude 9°10′51″ (E), altitude 255 m). Sown in plastic pots fitted with commercial peat and sand (1:2, *v*/*v*) and maintained under greenhouse conditions (naturally lit with sunlight, with a temperature range of 20–25 °C, relative humidity range of 50–80%). Seedlings were pre-cultivated for one month. Four replicates were performed. Nutrient solutions were renewed every 4 days. At the end of the treatment, plants were dried in the open air for 10 days and reduced to powder in order to be analyzed.

### 4.4. Microwave-Assisted Extraction

The extracting solvent (80/20 MeOH/water solution (*v*/*v*), 60 mL) was added to 2 g sample of plant material. at 60 °C for 10 min, with a power of 800 W. Samples were thoroughly cooled before opening the vessels, the extract separated by filtration and washed with MeOH (5 mL for three times). The extract was then treated with charcoal and polyvinylpyrrolidone (PVP). Briefly, charcoal (500 mg) was added to the methanolic extract under magnetic stirring at room temperature. After five minutes, charcoal was filtered off, solvent was evaporated to dryness under vacuum to 35 °C. The dried residue was then suspended in a solution of 70/30 acetone/water (*v*/*v*) (10 mL) and ethanol (10 mL) and added with 50 mg of PVP. After 15 min of mechanical stirring in an ice bath, the solvent was filtered and evaporated at reduced pressure, thus obtaining the crude extract (12.6 ± 0.7% yield). 

All dry extract obtained were stored at 4 °C until analysis. Each extraction experiment was carried out in triplicate.

### 4.5. Phytochemical Screening

#### 4.5.1. Determination of Total Phenolic Contents (TPC)

It was determined based on the method described by Mau et al. (2001) [58]. Briefly, 125 μL of extract was added to 500 µL deionized water and 125 µL of the Folin–Ciocalteu reagent. After shaking, the mixture was incubated for 3 min at room temperature. Then, 1250 µL of 7% Na_2_CO_3_ solution was added, the volume adjusted to 3 mL using distilled water, then the extract was mixed vigorously and held for 90 min at room temperature. Absorbance of mixtures was measured at 765 nm, against a blank. TPC of each individual sample was expressed as mg gallic acid equivalents (GAE)/g dry weight extract (DW). The analyses were conducted in triplicate and results are expressed as mean ± standard error (SE).

#### 4.5.2. Determination of Total Flavonoid Contents (TFC)

It was determined as described by Jia et al. [59] and Dewanto et al. [60]. 250 µL of the extract or standard solution of (+)-catechin was added to a 75 µL of a 5% NaNO_2_ solution. After 6 min, 150 µL of a 10% AlCl_3_ was added and allowed to stand for 5 min before 0.5 mL of 1 M NaOH was added. The mixture was brought to 2.5 mL with distilled water and thoroughly mixed. The absorbance was measured at 510 nm. Total flavonoid content was expressed as mg catechin/g dry weight extract (mg CE/g DW). 

#### 4.5.3. NMR Characterization and Quantification

All NMR spectra were recorded on an Agilent 400 MR DD2 VNMRJ™ Software Rev. 4.2 with a one NMR probe and a Premium Shielded 400/54 magnet.

For marrubiin characterization, spectra were acquired at 30 °C using 32,768 K data points and spectral width of 15.5 ppm and 240 ppm for ^1^H and ^13^C respectively. The 2D spectra used 1024 × 1024 data point matrices. Non shifted sine-bell window functions were used along F1 and F2 axes for gCOSY and gHMBC experiments. Proton—Solvent suppression with wet1D gCOSY—Pulse Field Gradient Correlation Spectroscopy (H-H) gHSQCAD—Pulse Field Gradient Heteronuclear Single Quantum Correlation Spectroscopy Adiabatic Version (C-H).The instrument was previously tested for ^1^H Line Shape, ^1^H Resolution, ^1^H Sensitivity and ^13^C Sensitivity, according to Agilent guidelines. 

For quantification assay, ^1^H spectra were acquired at 30 °C using 32,768 K data points, spectral width of 15.5 ppm and PW = 90°. The spectra were recorded without using solvent suppression, in order to avoid baseline artifacts that can interfere with a precise integration.

#### 4.5.4. HPLC-PAD/UV-CD and HPLC-ESI-MS/MS Analysis

The extracts were analyzed using both a high performance liquid chromatography-diode array system coupled on-line to a circular dichroism detector, and high performance liquid chromatography-electrospray-tandem mass spectrometry. Each sample was dissolved in acetonitrile (ACN) (25 mg/mL) and filtered with a 0.45 μm GH Polypro (GHP) membrane before injection into the HPLC-system.

The sample was analyzed on a XBridge Phenyl (150 × 4.6 mm i.d., 5 μm) column. The mobile phase is composed by Water (A) and ACN (B), both containing 0.1% (*v*/*v*) formic acid. The elution was performed in gradient conditions, starting from 10 to 90% of B in 20 min, followed by re-equilibration step of 8 min. The flow rate was 1 mL/min and the elution was performed at room temperature. The UV detection was fixed at 220 nm for HPLC-UV/PAD analysis and at 220, 254, 280 nm for HPLC-ESI-MS-MS analysis. 

Mass spectra were generated both in positive and in negative ion mode under constant instrumental conditions. For positive ion mode: ion spray voltage 4.5 kV, capillary voltage 7 V, capillary temperature 220 °C, and tube lens voltage 120 V. For negative ion mode: ion spray voltage 5 kV, capillary voltage −12 V, capillary temperature 220 °C, and tube lens voltage −60 V.

MS/MS data were acquired in Dependent scan mode (Full-scan MS followed by MS/MS of the most intense ion).

### 4.6. Antioxidant Activity

#### Free Radical Scavenging Activity

The free radical scavenging activity (FRS) of the extracts was determined by using a 2,2-diphenyl-1-picrylhydrazyl (DPPH) assay [37]. A commercially available standardized green tea extract (Green Select^®^) was used as a standard. Briefly, both dried extracts and the standard were dissolved in MeOH at a concentration of 250 μg/mL; stock solutions were then serially diluted in MeOH two-fold. The reaction mixture was prepared by adding 100 µL of each extract solution (or standard solution) to 3.9 mL of DPPH solution, freshly prepared by dissolving DPPH in methanol/KH_2_PO_4_ and NaOH buffer (50/50 *v*/*v*) at a concentration of 6 × 10^−5^ M, giving test solutions with final concentrations of 2.5, 5, 10, 20, 40, 80, 100 and 120 μg/mL. After 20 min of incubation at room temperature, the absorbance was measured at 515 nm by the UV-Visible.

FRS was expressed as a percent compared with the control, consisting of 3.9 mL of DPPH solution and 100 µL of methanol. The percent inhibition of the DPPH radical by the test solution was calculated using the following formula:

FRS%= [(Abs control − Abs sample)/Abs control] × 100

The analyses were carried out in triplicate and results are expressed as mean ± SE. IC_50_ values were calculated by using Graph Pad Prism 4.0.

### 4.7. Wound Healing Activity

#### 4.7.1. Cytoxicity Test towards Human Dermal Fibroblasts

NHDF fibroblasts (juvenile fibroblast from foreskin; Promocell GmbH, Heidelberg, Germany) from 9th to 11th passage were used. Cells were cultured in a polystyrene flask (Greiner Bio-One, PBI International, Milan, Italy) with complete medium (CM), consisting of Dulbecco’s Modification of Eagle’s Medium (DMEM), supplemented with 1% (*v*/*v*) antibiotic solution (100×) (Penicillin, Streptomycin, Amphotericin B) and 10% (*v*/*v*) of fetal bovine serum (FBS). Cells were maintained in incubator (Shellab^®^ Sheldon^®^ Manufactoring Inc., Cornelius, OR, USA) at 37 °C with 95% air and 5% CO_2_ atmosphere. All the operations required for cell culture were carried out in a vertical laminar air flow hood (Ergosafe Space 2, PBI International, Milan, Italy). After the cells had reached 80–90% confluence in the flask (about one week), they were washed with D-PBS (Dulbecco’s Phosphate Buffer Solution) and then collected by trypsinization using 3 mL of Trypsin-EDTA solution 0.25% (*v*/*v*) for 5 min at room temperature, followed by the addition of 7 mL of CM to stop the proteolytic action of trypsin and to facilitate cell detachment. Afterwards, cell suspension was centrifuged (TC6, Sorvall Products, Newtown, CT, USA) at 112 g for 10 min. The supernatant was discarded and the cells were resuspended in 6 mL of serum-free medium (*M*_w_/s). The amount of cells in suspension was determined in a counting chamber (Hycor Biomedical, Garden Grove, CA, USA) using 0.5% (*w*/*v*) trypan blue solution to visualize and count viable cells. Cells were seeded in a 96-well plate at a density of 6 × 104 cells/well (0.35 cm^2^ area) in 200 μL of CM and allowed to grow for 24 h. Then, the medium was removed and cells were placed in contact for 24 h with 200 μL of extracts diluted in CM. The following dilutions were considered: 500 µg/mL (1); 250 µg/mL (2); 125 µg/mL (3); 50 µg/mL (4); 5 µg/mL (5). After 24 h contact, the cytotoxicity was evaluated by MTT test. In particular, samples were removed and 50 μL of MTT (3-(4,5-dimethylthiazol-2-yl)-2,5-diphenyltetrazolium bromide) 8 μM in 100 μL of HBSS (pH 7.4) were added to each well and incubated for 4h at 37 °C. Finally, DMSO was added to dissolve formazan crystals. CM alone or in mixture with DMSO (same concentration as in extracts) was used as control. Absorbance measurements were performed employing a microplate spectrophotometer (IMark^®^ Microplate reader, Bio-Rad Laboratories Srl, Segrate, Milan, Italy) at 570 nm.

#### 4.7.2. Wound Healing Test towards Human Dermal Fibroblasts

Such test is based on the employment of Petri μ-Dish (Ibidi, Giardini, Milan, Italy) in which an insert is enclosed. The insert is formed of 2 chambers with a growth area of 0.22 cm^2^ divided by a septum with a width of cell free gap of 500 µm ± 50 µm. Normal Human Dermal Fibroblasts (NHDF) (PromoCell GmbH, Heidelberg, Germany) from 9th and 11th passage were seeded in each chamber at 105 cells/cm^2^ and growth at confluence in standard conditions as previously described. After 24 h cells reach confluence and the insert is removed displaying 2 areas of cell substrates divided from the prefixed gap. Cell substrates were put in contact with 500 µL of sample. At predetermined times (0, 24, 48, 72 h). The extent of cell migration was photographed and measured using inverted optical microscope (Leica DMI 3000B) with internal camera programmed for Leica Application Suite EZ V 1.8.0 and V 1.8.1 PC (Leica Microequipments, Milano, Italy).

### 4.8. Statistical Analysis

All data are expressed as the means ± standard deviation of three replicates. Significant differences among different treatments were calculated using the one-way analysis of variance (ANOVA), followed by Dancan’s multiple-range test at 5% level (*p* ≤ 0.05). All analyses were performed using SPSS v19.0 software (Chicago, IL, USA) for Windows.

## 5. Conclusions

In the current study, we demonstrated the potential of *Marrubium vulgare* L. leaves methanolic extract in wound repair. The MASE extraction procedure allows to obtain a *Marrubium vulgare* hydro-alcoholic extract rich (6.62%) in marrubiin (determined by quantitative NMR) and characterized by a high content of phenols and flavonoids (colorimetric assays), and several phenylethanoid glycosides (HPLC-UV/PAD-ESI/MS). Such extract showed antioxidant and wound healing properties promoting the cell migration and proliferation of fibroblasts. This is the first study proving the beneficial effect of *Marrubium vulgare* L. leaves extract on wound healing. Further studies aimed at identifying the compounds responsible for its wound healing activities are ongoing.

## Figures and Tables

**Table 1:** ^1^H and ^13^C assignment of marrubiin.

^1^H	δ (ppm)	^13^C	δ (ppm)
**1α**	1.82	**1**	28.32
**1β**	1.31	**2**	17.78
**2α**	1.72	**3**	28.09
**2β**	1.55	**4**	44.02
**3α**	1.44	**5**	44.79
**3β**	2.05	**6**	77.53
**5**	2.31	**7**	31.03
**6**	4.84 ddd	**8**	31.72
**7α**	1.77	**9**	75.52
**7β**	2.13	**10**	39.63
**8**	2.13	**11**	35.50
**11A**	1.77	**12**	20.65
**11B**	1.83	**13**	125.56
**12A**	2.52	**14**	110.42
**12B**	2.52	**15**	142.69
**14**	6.31 dd	**16**	138.29
**15**	7.38 t	**17**	15.58
**16**	7.27 ddt	**18**	21.93
**17**	0.97 s	**19**	185.98
**18**	1.29 s	**20**	21.93
**20**	1.03 s		

**Table 2:** Results obtained from ESI/MS/MS experiments.

Retention Time (min)	Negative Ions	Fragment Ions	Positive Ions	MW	Fragment Ions	Structure	Compound Name
5.58	887.27 [M − H]^−^	725.16 [M − H-162]^−^ 593.18 [M − H-162-132]^−^	906.02 [M + NH_4_]^+^	888	889.12 [M + NH_4_-NH_3_]^+^ 624.71 [M + NH_4_-NH_3_-132-132]^+^ 478.60 [M + NH_4_-NH_3_-132-132-146]^+^ 470.68 [M + NH_4_-NH_3_-132-132-154]^+^ 324.90 [M + NH_4_-NH_3_-132-132-146-154]^+^	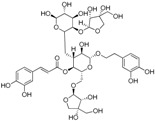	Marruboside
5.79	755 [M − H]^−^	593.18 [M − H-162]^−^ 461.16 [M − H-162-132]^−^ 447.14 [M − H-162-146]^−^	773 [M + NH_4_]^+^	756	756.50 [M + NH_4_-NH_3_]^+^ 624.83 [M + NH_4_-NH_3_-132]^+^ 478.58 [M + NH_4_-NH_3_-132-146]^+^ 470.68 [M + NH_4_-NH_3_-132-154]^+^ 324.82 [M + NH_4_-NH_3_-132-154-146]^+^	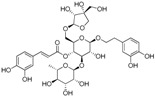	Forsythoside B
6.17	755 [M − H]^−^	593.18 [M − H-162]^−^ 461.16 [M − H-162-132]^−^	773 [M + NH_4_]^+^	756	756.50 [M + NH_4_-NH_3_]^+^ 624.83 [M + NH_4_-NH_3_-132]^+^ 478.58 [M + NH_4_-NH_3_-132-146]^+^ 470.68 [M + NH_4_-NH_3_-132-154]^+^ 324.82 [M + NH_4_-NH_3_-132-154-146]^+^	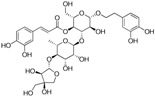	Samioside
6.36	623.18 [M − H]^−^	461.16 [M − H-162-132]^−^ 315.0 [M − H-162-132-146]^−^	641.89 [M + NH_4_]^+^	624	624.71 [M + NH_4_-NH_3_]^+^ 478.51 [M + NH_4_-NH_3_-146]^+^ 470.54 [M + NH_4_-NH_3_-154]^+^ 324.82 [M + NH_4_-NH_3_-154-146]^+^	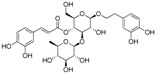	Verbascoside
13.07	ND	ND	336.66 [M + H]^+^	336	319.14 [M + H-H_2_O]^+^ 301.1 [M + H-2H_2_O]^+^ 289.1 [M + H-H_2_O-H_2_C=O]^+^	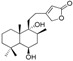	Deacetylvitexilactone
14.49	ND	ND	665.24 [2M + H]+ 1016.8 [6M + H + K]+	332	ND	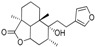	Marrubiin

## Supplementary Materials

The following are available online, Figure S1: ^1^H-NMR spectrum of marrubiin, Figure S2: ^13^C-NMR spectrum of Marrubiin, Figure S3: COSY spectrum of marrubiin, Figure S4: HSQC spectrum of marrubiin, Figure S5: MS/MS spectrum negative ions of compound with retention time (t_R_) of 5.58 min, parent ion *m*/*z* 887.17, Figure S6: MS/MS spectrum positive ions of compound with retention time (t_R_) of 5.58, parent ion *m*/*z* 906.00, Figure S7: MS/MS spectrum negative ions of compound with retention time (t_R_) of 5.79, parent ion *m*/*z* 755.22, Figure S8: MS/MS spectrum positive ions of compound with retention time (t_R_) of 5.79, parent ion *m*/*z* 773.92, Figure S9: MS/MS spectrum negative ions of compound with retention time (t_R_) of 6.16 parent ion *m*/*z* 755.22, Figure S10: MS/MS spectrum positive ions of compound with retention time (t_R_) of 6.16 parent ion *m*/*z* 773.92, Figure S11: MS/MS spectrum negative ions of compound with retention time (t_R_) of 6.36, parent ion *m*/*z* 623.16, Figure S12: MS/MS spectrum positive ions of compound with retention time (t_R_) of 6.36, parent ion *m*/*z* 641.89.
