# Supplementary material for: Marrubium vulgare L. Leave Extract: Phytochemical Composition, Antioxidant and Wound Healing Properties
Source: Molecules. 2017 Oct 28;22(11):1851. doi: 10.3390/molecules22111851 (PMC6150401; doi:10.3390/molecules22111851)
Supplement: Supplementary file 1 [file molecules-22-01851-s001.pdf]

Supplementary Materials: *Marrubium vulgare* L. Leave Extract: Phytochemical Composition, Antioxidant and Wound Healing Properties

Bédis Amri [1], Emanuela Martino [2,3,*], Francesca Vitulo [4], Federica Corana [5], Leila Bettaieb-Ben Kaâb [1], Marta Rui [6], Daniela Rossi [6], Michela Mori [6], Silvia Rossi [3,6] and Simona Collina [3,6,*]

[1] Département de Biologie, Faculté des Sciences de Tunis el Manar, Unité de recherche 'Nutrition et métabolismes azoté et protéines de stress' (99 UR/09-20), 1002 Tunis, Tunisia; bedisamri@gmail.com (B.A.); leila.bk@planet.tn (L.B.-B.K.)

[2] Department of Earth and Environmental Sciences, University of Pavia, Via S. Epifanio 14, 27100 Pavia, Italy

[3] Centre for Health Technologies (CHT), University of Pavia, Viale Taramelli 12, Pavia 27100, Italy; silvia.rossi@unipv.it

[4] Indena S.p.A., Via Don Minzoni, 6, 20090 Settala, Italy; francesca.vitulo@indena.com

[5] Centro Grandi Strumenti (CGS), University of Pavia, Via Bassi 21, 27100 Pavia, Italy; federica.corana@unipv.it

[6] Department of Drug Sciences, Medicinal Chemistry section, University of Pavia, Viale Taramelli 12, 27100 Pavia, Italy; marta.rui01@universitadipavia.it (M.R.); daniela.rossi@unipv.it (D.R.); michela.mori@unipv.it (M.M.)

* Correspondence: emanuela.martino@unipv.it (E.M.); simona.collina@unipv.it (S.C.); Tel.: +39-0382-987-379 (S.C.)

Table of Contents

1. NMR analysis………………………………………………………………………………………….. S2

2. MS experiments…………………………………………………………………………………………...S4

1. NMR analysis

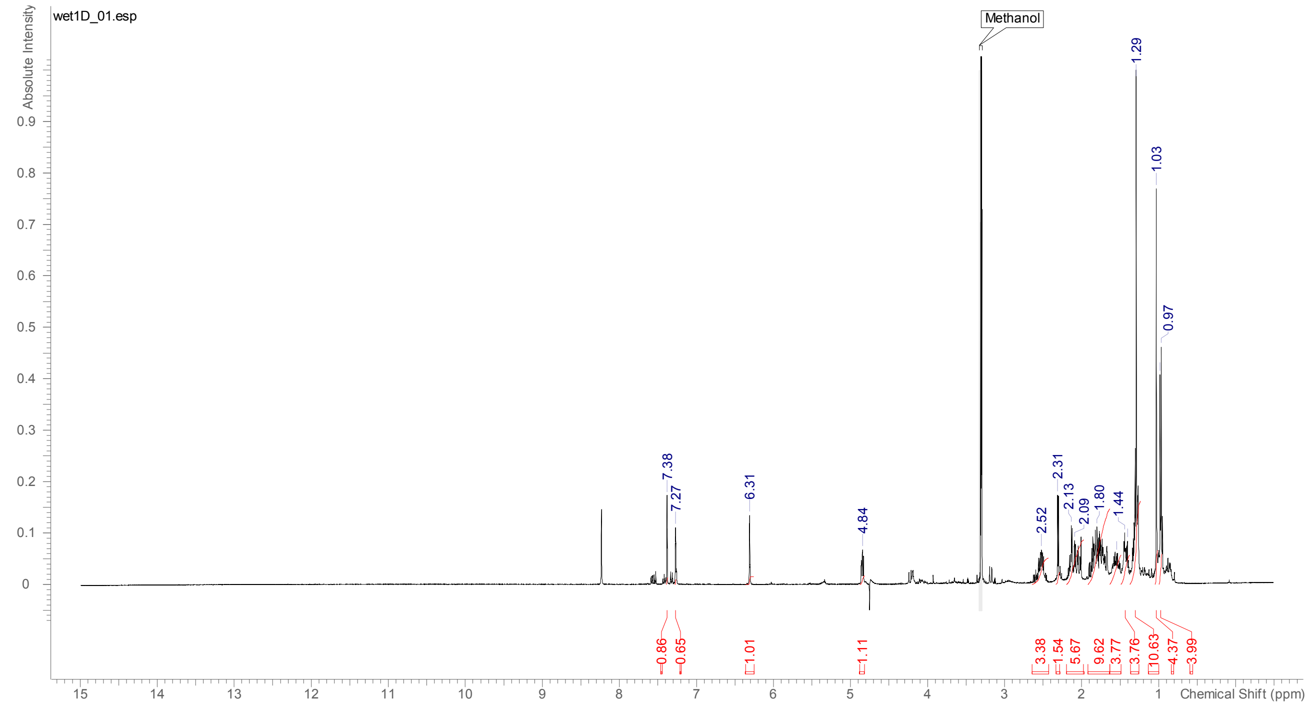

Figure S1. ^{1}H spectrum of marrubiin.

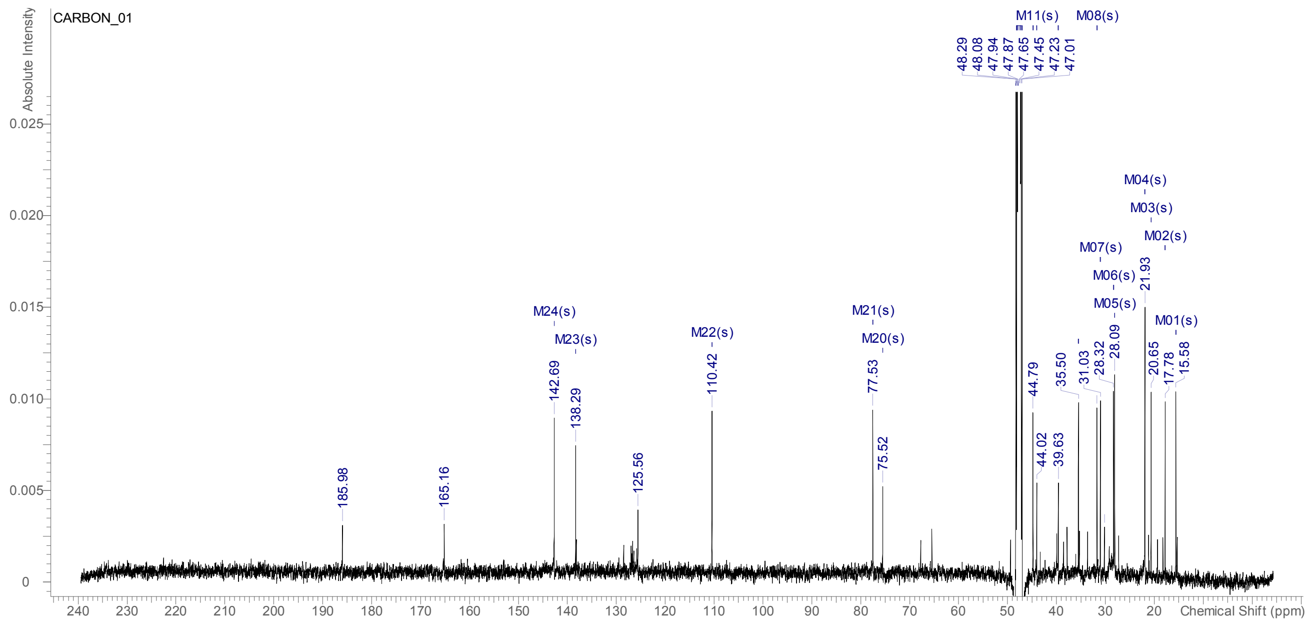

Figure S2. ^{13}C spectrum of Marrubiin.

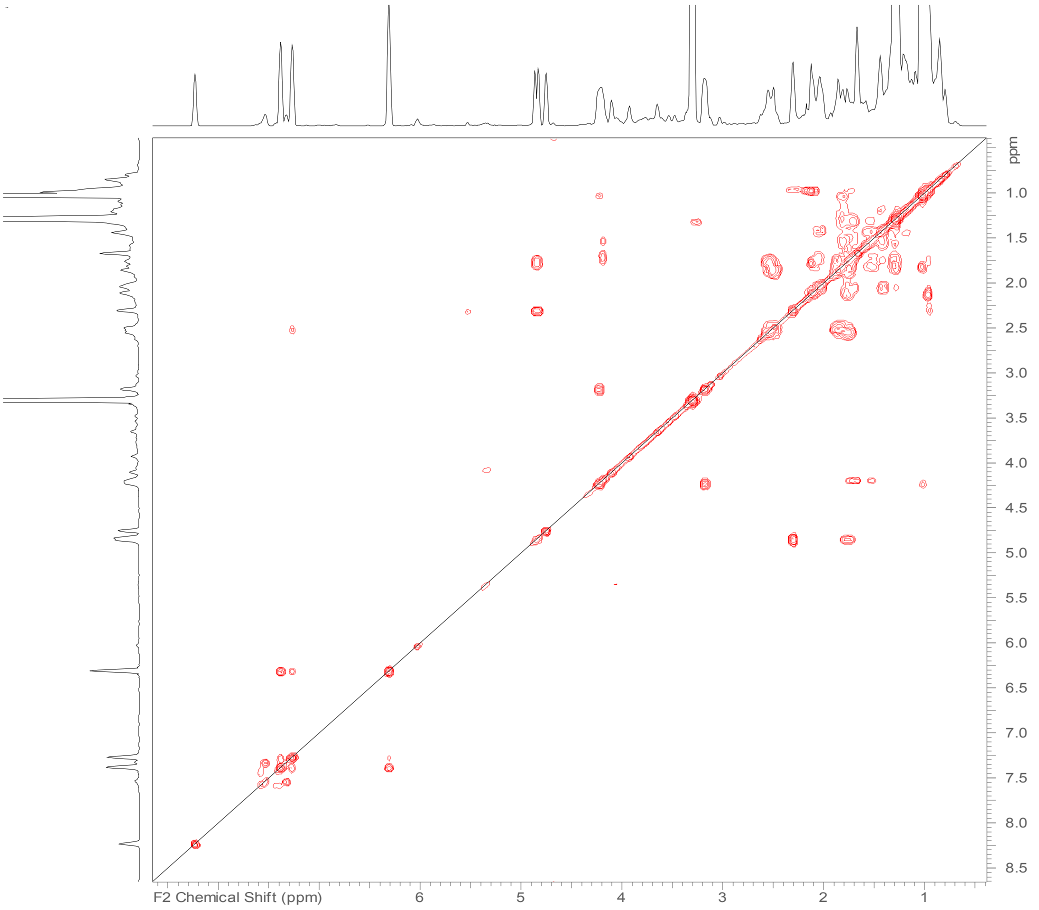

Figure S3. COSY spectrum of marrubiin.

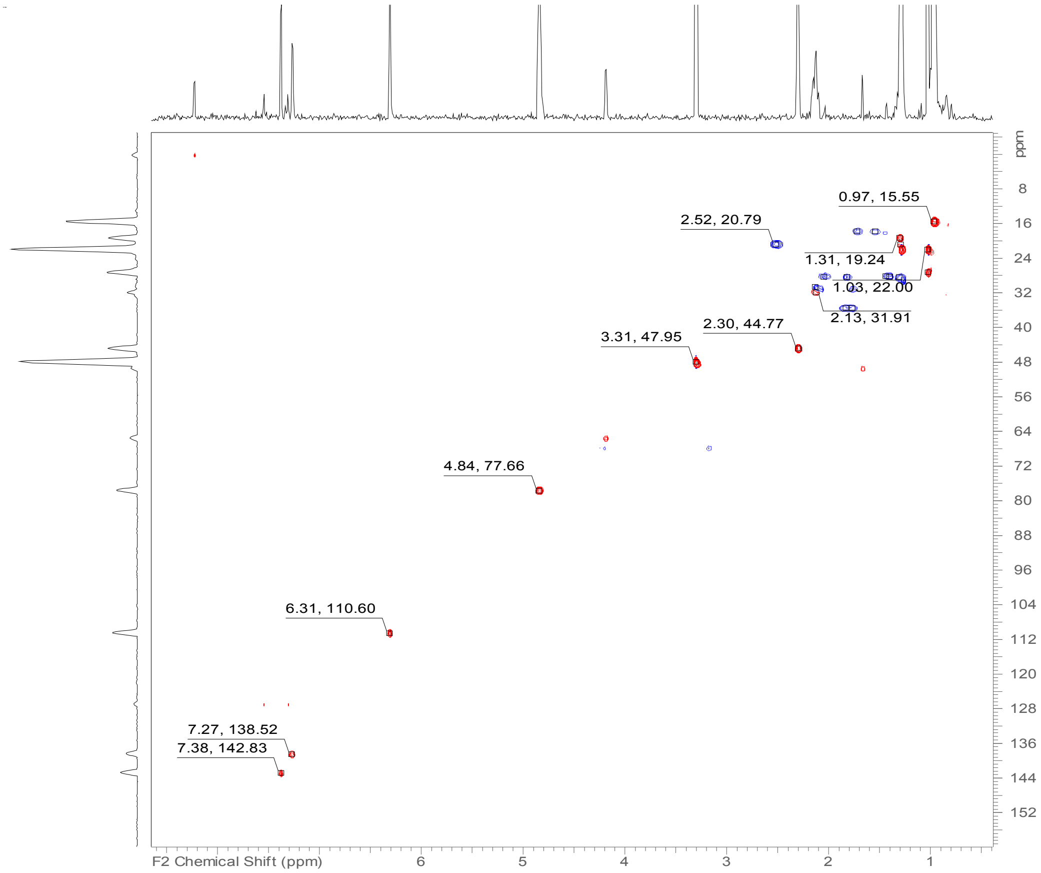

Figure S4. HSQC spectrum of marrubiin.

2. MS experiments

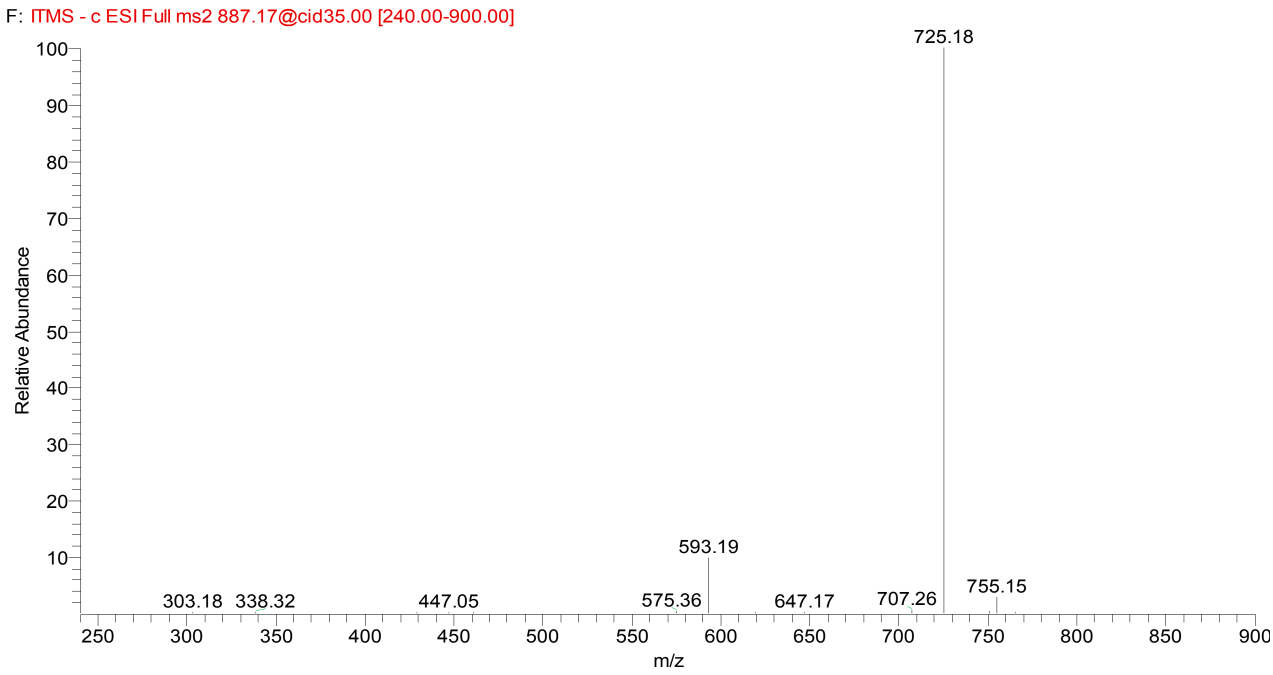

Figure S5. MS/MS spectrum negative ions of compound with retention time (t_R) of 5.58 min, parent ion m/z 887.17.

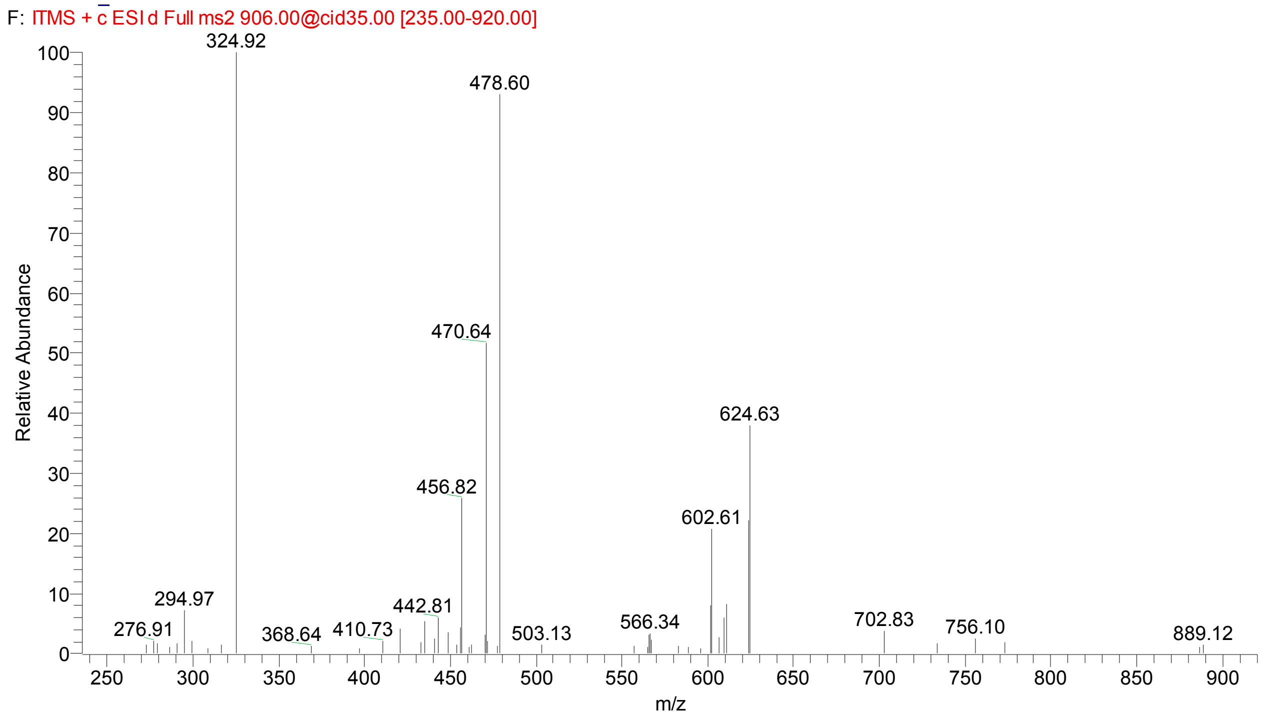

Figure S6. MS/MS spectrum positive ions of compound with retention time (t_R) of 5.58, parent ion m/z 906.00.

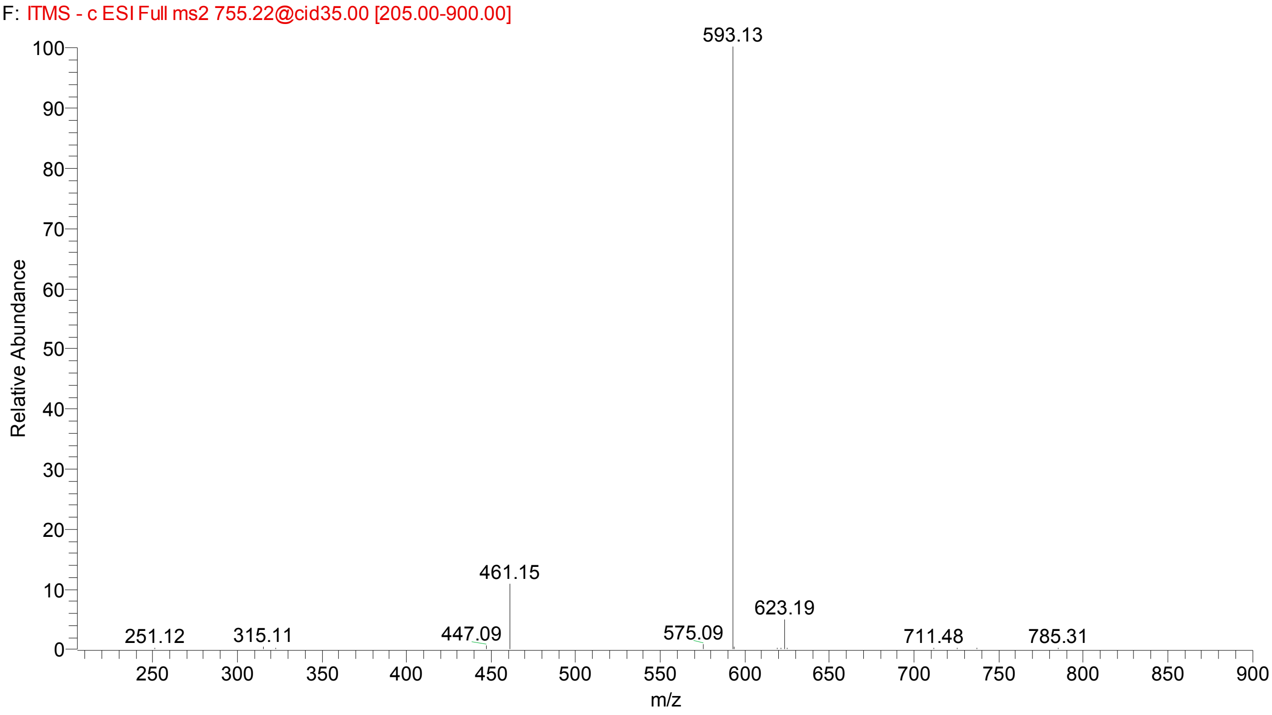

Figure S7. MS/MS spectrum negative ions of compound with retention time (t_R) of 5.79, parent ion m/z 755.22.

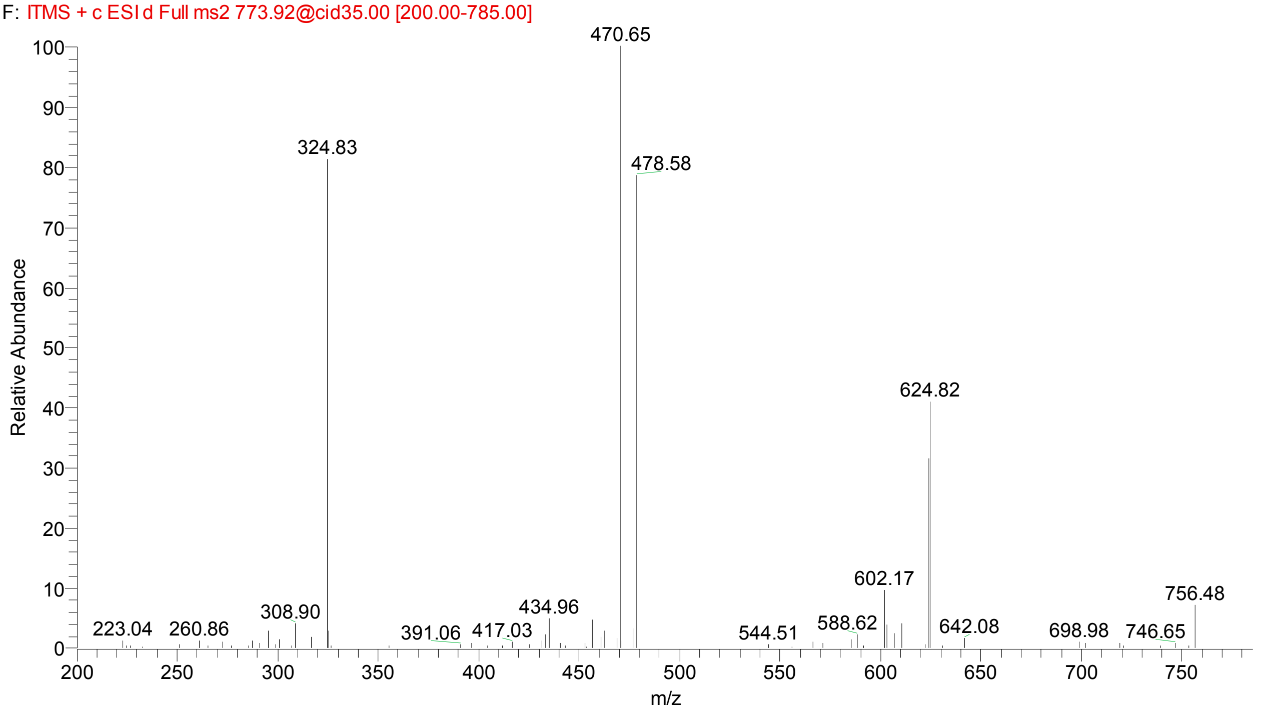

Figure S8. MS/MS spectrum positive ions of compound with retention time (t_R) of 5.79, parent ion m/z 773.92.

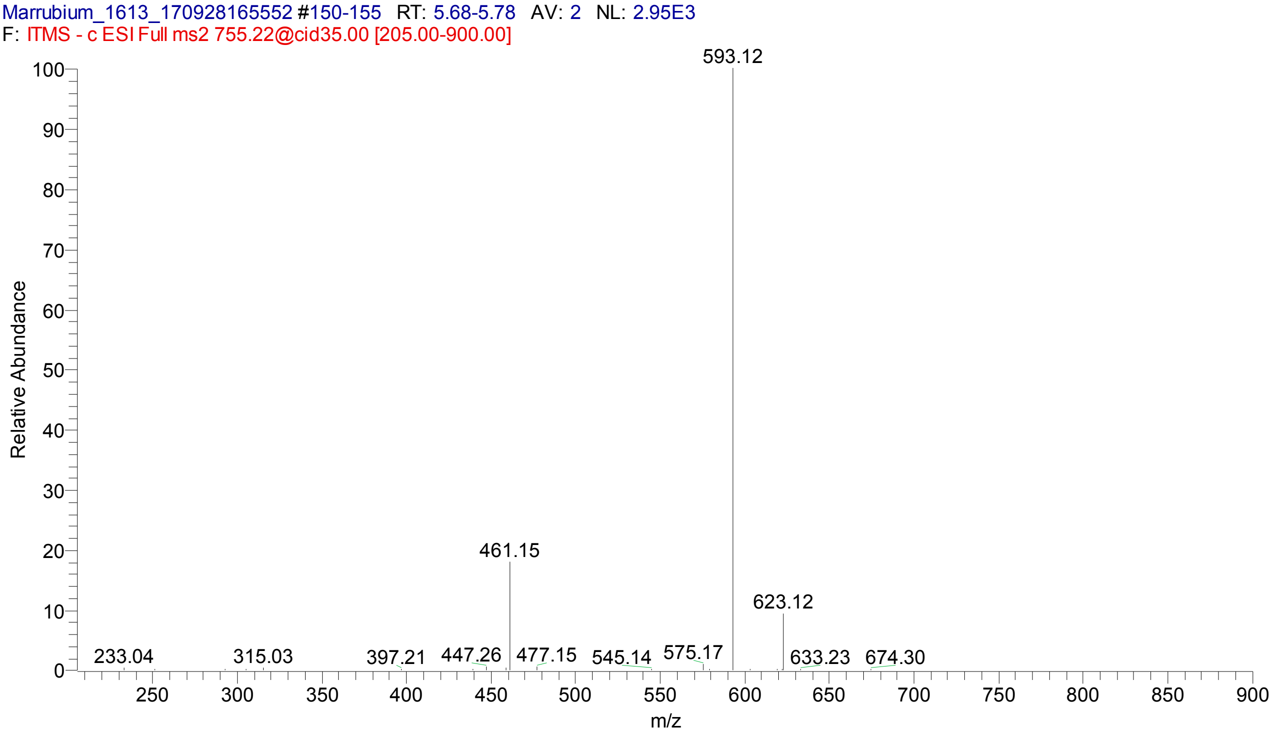

Figure S9. MS/MS spectrum negative ions of compound with retention time (t_R) of 6.16 parent ion m/z 755.22.

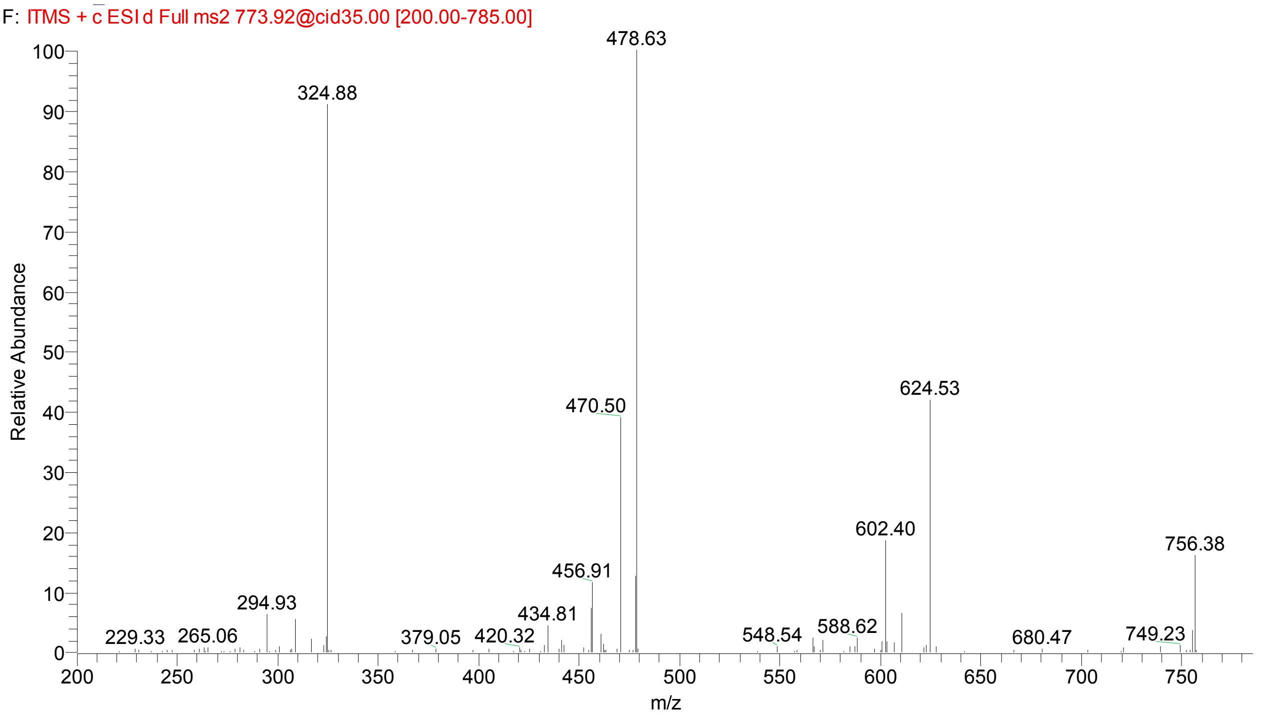

Figure 10. MS/MS spectrum positive ions of compound with retention time (t_R) of 6.16 parent ion m/z 773.92.

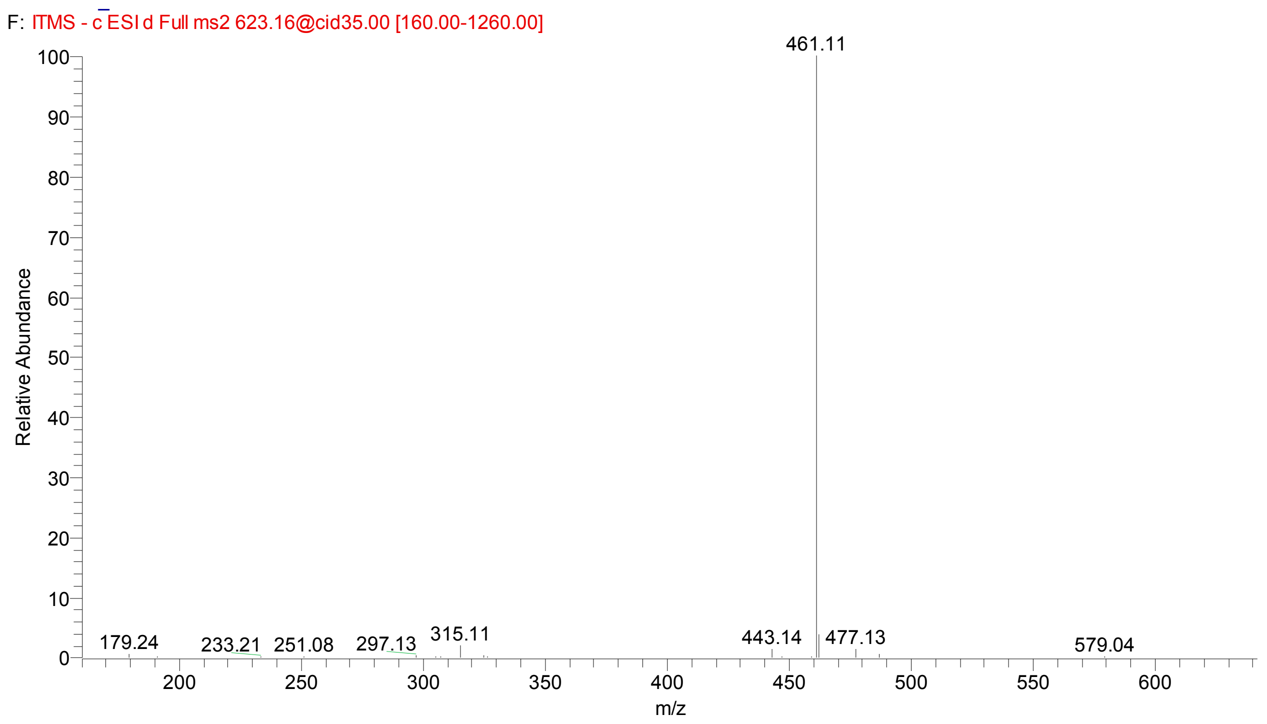

Figure S11. MS/MS spectrum negative ions of compound with retention time (t_R) of 6.36, parent ion m/z 623.16.

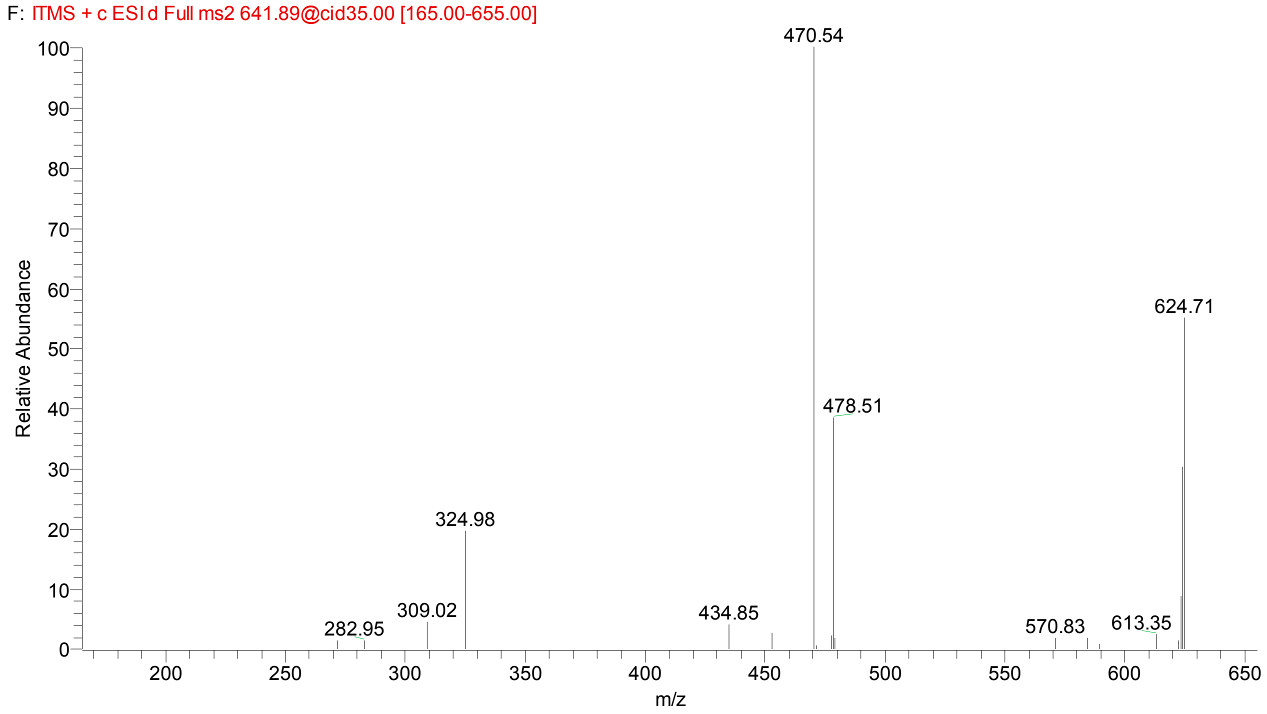

Figure S12. MS/MS spectrum positive ions of compound with retention time (t_R) of 6.36, parent ion m/z 641.89.
